# DSC Perfusion MRI Artefact Reduction Strategies: A Short Overview for Clinicians and Scientific Applications

**DOI:** 10.3390/jcm14134776

**Published:** 2025-07-06

**Authors:** Chris W. J. van der Weijden, Ingomar W. Gutmann, Joost F. Somsen, Gert Luurtsema, Tim van der Goot, Fatemeh Arzanforoosh, Miranda C. A. Kramer, Anne M. Buunk, Erik F. J. de Vries, Alexander Rauscher, Anouk van der Hoorn

**Affiliations:** 1 Department of Nuclear Medicine and Molecular Imaging, Medical Imaging Center, University of Groningen, 9713 GZ Groningen, The Netherlands; 2 Department of Radiology, Medical Imaging Center, University Medical Center Groningen, University of Groningen, 9713 GZ Groningen, The Netherlands; 3 Physics of Functional Material, Faculty of Physics, University of Vienna, 1090 Vienna, Austria; 4 Department of Radiation Oncology, University Medical Center Groningen, University of Groningen, 9713 GZ Groningen, The Netherlands; 5 Department of Neurology, University Medical Center Groningen, University of Groningen, 9713 GZ Groningen, The Netherlands; 6 Department of Paediatrics, University of British Columbia, Vancouver, BC V6T 1Z4, Canada; 7 UBC MRI Research Centre, University of British Columbia, Vancouver, BC V6T 1Z4, Canada; 8 Department of Physics and Astronomy, The University of British Columbia, Vancouver, BC V6T 1Z4, Canada

**Keywords:** dynamic susceptibility contrast, perfusion weighted imaging, artefacts, pre-processing

## Abstract

MRI perfusion is used to diagnose and monitor neurological conditions such as brain tumors, stroke, dementia, and traumatic brain injury. Dynamic Susceptibility Contrast (DSC) is the most widely available quantitative MRI technique for perfusion imaging. Even in its most basic implementation, DSC MRI provides critical hemodynamic metrics like cerebral blood flow (CBF), blood volume (CBV), mean transit time (MTT), and time between the peak of arterial input and residue function (Tmax), through the dynamic tracking of a gadolinium-based contrast agent. Notwithstanding its high clinical importance and widespread use, the reproducibility and diagnostic reliability are impeded by a lack of standardized pre-processing protocols and quality controls. A comprehensive literature review and the authors’ aggregated experience identified common DSC MRI artefacts and corresponding pre-processing methods. Pre-processing methods to correct for artefacts were evaluated for their practical applicability and validation status. A consensus on the pre-processing was established by a multidisciplinary team of experts. Acquisition-related artefacts include geometric distortions, slice timing misalignment, and physiological noise. Intrinsic artefacts include motion, B_1_ inhomogeneities, Gibbs ringing, and noise. Motion can be mitigated using rigid-body alignment, but methods for addressing B_1_ inhomogeneities, Gibbs ringing, and noise remain underexplored for DSC MRI. Pre-processing of DSC MRI is critical for reliable diagnostics and research. While robust methods exist for correcting geometric distortions, motion, and slice timing issues, further validation is needed for methods addressing B_1_ inhomogeneities, Gibbs ringing, and noise. Implementing adequate mitigation methods for these artefacts could enhance reproducibility and diagnostic accuracy, supporting the growing reliance on DSC MRI in neurological imaging. Finally, we emphasize the crucial importance of pre-scan quality assurance with phantom scans.

## 1. Introduction

Dynamic Susceptibility Contrast (DSC) perfusion MRI is routinely used for diagnosis and monitoring of various neurological disorders, like brain tumors, stroke, cerebrovascular diseases, neurodegenerative diseases, and traumatic brain injury [1,2,3]. For brain tumors, DSC MRI is often used to differentiate between tumor types, grading tumors, treatment planning, and monitoring of the response to therapy [4]. In stroke, DSC MRI is used to identify tissue at risk with impaired perfusion surrounding infarctions (penumbra) versus tissue that is already necrotic, which is pivotal for treatment decision making (e.g., thrombolysis, thrombectomy). For neurodegenerative diseases, DSC MRI might help in distinguishing vascular dementia from other types, like Alzheimer’s disease, by showing areas of reduced perfusion [5].

**Figure 1:**
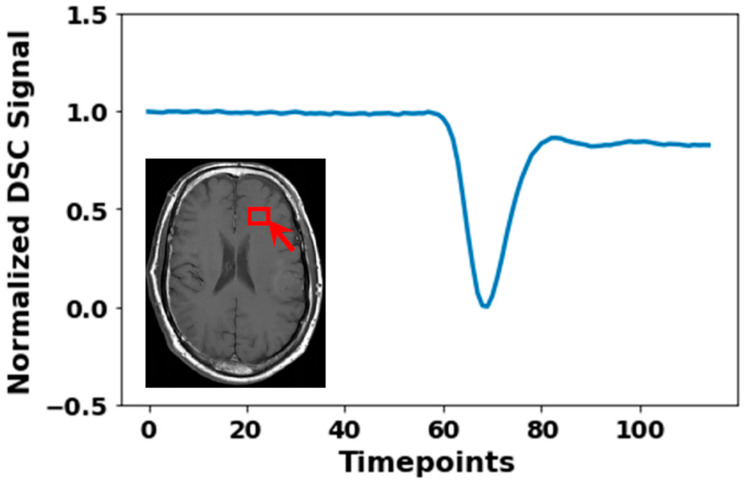
Normalized Dynamic Susceptibility Contrast (DSC) MRI signal of a 3 × 3 × 3 mm^3^ cube region located in healthy brain tissue (region indicated by the red box). Normalization was the division of observed signal intensity by max observed signal intensity.

Giving a basic overview of the DSC MRI method, DSC MRI uses an echo planar imaging (EPI) sequence to track a bolus of paramagnetic contrast agent over time [6,7]. The acquisition of DSC MRI starts prior to the injection of the contrast agent, to obtain baseline images unaffected by the contrast. Due to the paramagnetic properties of the contrast agent, a drop in T_2_*w and T_2_w signal intensity is observed when the contrast agent passes through the tissue (Figure 1). The paramagnetic properties of the contrast agent increase local magnetic field inhomogeneities. This leads to faster dephasing of transverse magnetization, resulting in a loss of signal intensity during contrast bolus passage. As the contrast agent consists of large hydrophilic molecules (547 Da), they cannot pass through the blood–brain barrier (BBB) and hence the contrast agent resides in blood [8,9]. Therefore, the change in signal intensity after injection of contrast agent as detected with DSC MRI, can be used to obtain indices like mean transit time (MTT), cerebral blood volume (CBV), and cerebral blood flow (CBF), and time between the peak of arterial input and residue function (Tmax), which reflect the hemodynamic properties of the tissue through which the contrast travels [6,7,10,11]. However, this assumption that contrast agents are not able to cross the BBB does not hold for all conditions. For instance, brain tumors often have a leaky BBB, resulting in contrast agent leakage into the extracellular space [12,13]. To maintain the accuracy of parameter estimations, a leakage correction can be performed. Furthermore, parameter estimations can be affected by image artefacts, which could impact the diagnostic value of the images.

While perfusion MRI is considered a quantitative MRI method, a standardized protocol for the pre-processing of DSC MRI data to mitigate image artefacts has not yet been developed. This might severely impact the reproducibility of findings between studies, but also affect the diagnosis of patients or cause erroneous scientific findings. In contrast to the lack of standardized pre-processing methods for DSC MRI, for other quantitative MRI methods, like diffusion MRI, multiple publications have been released that describe the most suitable diffusion MRI pre-processing [14,15,16]. Hence, there is a need for the standardization of the pre-processing of DSC MRI. Pre-processing in the context of this manuscript is the execution of techniques/corrections aimed at improving image quality by correcting for artefacts related to data acquisition. Therefore, this study is a narrative review aimed to provide a first structured overview of common DSC image artefacts and the validation status of the artefact correction techniques for brain DSC MRI.

## 2. Methods

The literature was reviewed for common sources of artefacts in DSC MRI and potential solutions to overcome them using PubMed, focussing on (1) DSC MRI artefacts, including motion, geometric distortion, B_1_ field inhomogeneity, Gibbs ringing, slice-timing misalignment, noise, and physiological noise, (2) DSC pre-processing algorithms for the respective artefacts, and (3) EPI related artefacts, irrespective of the specific sequence. DSC MRI typically makes use of an EPI sequence to enable rapid image acquisition; therefore, the literature on EPI-related artefacts was considered, with a special emphasis on articles describing guidelines related to pre-processing of functional MRI (fMRI) and diffusion-weighted imaging (DWI).

For each type of artefact, the corresponding correction methods and pre-processing tools were identified. Subsequently, their practical applicability and validation status were assessed. Finally, a consensus with respect to proper DSC pre-processing methods was established. The expert consensus was established through collaboration among a multidisciplinary panel comprising physicists, neuroimaging scientists, neuropsychologists, neuroradiologists, and neuroscientists. Eleven experts contributed to the evaluation of artefact types in DSC MRI and the corresponding correction strategies, which were assessed based on their practical applicability and validation status in the literature. The process involved a systematic review and structured discussions to identify and agree upon optimal pre-processing approaches. Disagreements were resolved through iterative feedback and evidence-based reasoning, with priority given to methods supported by empirical validation. Consensus was reached when all expert domains aligned on the recommended artefact correction strategies for both clinical and scientific applications.

## 3. Results

DSC MRI typically utilizes 2D or 3D EPI sequences [6,7]. These sequences are most commonly performed with Gradient-Recalled-Echo (GRE) imaging to track the dynamic signal changes induced by the contrast bolus. For some advanced applications, combined GRE-SE techniques are used [17,18]. Due to use of EPI sequences and intrinsic MRI properties, DSC MRI suffers from a wide variety of artefacts (Table 1), which can be either mitigated by ensuring proper data acquisition i.e., by tuning sequence parameters like echo-time (TE) or by employing sophisticated algorithms on the images to correct for the artefacts, which is called pre-processing. These artefacts may require pre-processing and include geometric distortions, slice-timing misalignment, image intensity variations, Gibbs ringing, subject motion, physiological artefacts, and noise.

Not all artefacts affect DSC MRI data to the same extent; for example, Gibbs ringing is typically less severe than motion artefacts or geometric distortions, which are considered the most prominent sources of error in DSC MRI. However, it is important to emphasize that artefacts deemed less severe, as in less visible artefacts, are not necessarily less impactful. Even subtle image degradation can introduce systematic biases in parameter estimation, potentially leading to clinically relevant over- or underestimation of perfusion metrics. Such inaccuracies may influence diagnostic interpretation or prognostic assessment, underscoring the need for careful artefact identification and correction, regardless of perceived severity.

### 3.1. Geometric Distortions (B_0_)

**Figure 2:**
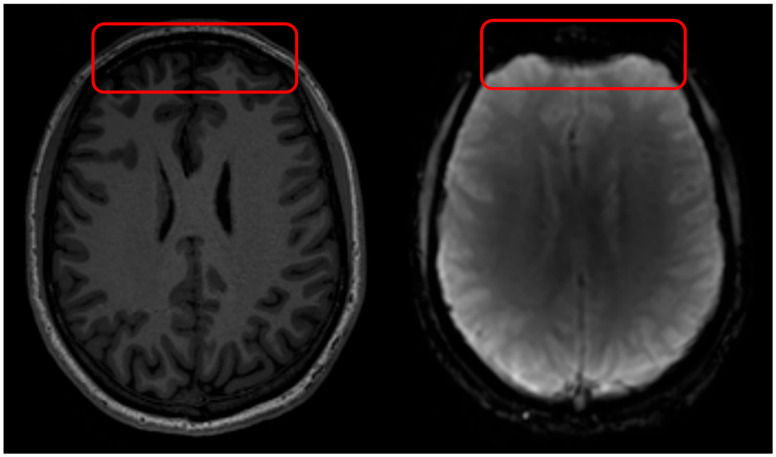
Geometric distortions in DSC MRI appear as misshapen or incorrectly sized brain structures, leading to spatial misregistration with anatomical images, primarily in regions near air/tissue interfaces. Geometric distortions in DSC MRI arise due to B_0_ field inhomogeneities and tissue susceptibility differences. On the left is a T_1_w MRI scan and on the right a DSC MRI scan with visible geometric distortions. The red square indicates tissue hyper intensities due to B_1_ field inhomogeneities.

Geometric distortions in DSC MRI result in misshapen or incorrectly sized brain structures, leading to spatial misregistration, causing the observed anatomical locations to deviate from their true anatomical positions (Figure 2). This can result in anatomic alterations observed in DSC MRI that do not correspond with the actual location of pathological tissue. Geometric distortions in EPI sequences commonly arise close to regions where the magnetic tissue properties change abruptly (like air, bone, and brain tissue), which impact the static B_0_ field [19,20,21,22,23]. This type of geometric distortion typically appears as deformations in the phase-encoding direction, most prominent near air–tissue interfaces such as the sinus areas in the brain. Geometric distortions can make certain areas in the brain appear in incorrect locations, have distorted shapes, or as ‘signal pile-up’ where signal from multiple voxels are displaced into a single voxel, causing localized signal increase, which complicates accurate brain mapping, diagnosis, or surgical planning.

For correcting the geometric distortions in the phase-encoding direction in EPI data, a B_0_ field map is required. The generation of a B_0_ field map is commonly estimated by acquiring images with an inverted phase encoding direction prior to the start of the DSC acquisition. Acquisition of images in opposing phase-encoding directions, while keeping all other parameters the same in regard to the DSC MRI acquisition, enables the estimation of a B_0_ field distortion map under the assumption that the distortions mirror symmetrical [19,20,24]. The geometric distortions can be estimated and corrected using the FSL “topup” correction. By comparing the opposing phase encoding images, Topup estimates the underlying field-induced displacement map, which describes how much each voxel is misregistered due to local B_0_ variations [19,20]. Once this displacement field is calculated, Topup applies an inverse warping function, realigning the affected voxels and correcting for geometric distortions. Alternatively, the classical approach of acquiring a separate B_0_ field map can be used to correct geometric distortions in the DSC MR images [24]. This could be straightforwardly performed using the “Field Map toolbox” in SPM [19,23,25]. The correction process begins with the acquisition of two separate GRE sequences, each with a single echo and different echo times (TE1 and TE2). By computing the phase difference between these images, a B_0_ field map is created, representing the local magnetic field variations. Next, the B_0_ field map is converted into a voxel displacement map, which estimates how much each voxel has shifted due to magnetic field inhomogeneities. Since EPI sequences are particularly vulnerable to distortions along the phase-encoding direction, this displacement map provides a precise correction factor for realigning affected voxels [19,23]. The Field Map Toolbox in SPM then applies an inverse warping function, which spatially corrects the distorted DSC MRI images by adjusting their voxel positions according to the displacement estimates. This ensures that the perfusion data aligns accurately with anatomical structures. In short, either a B_0_ field map obtained prior to injection of the contrast agent or a reversed phase-encoding direction DSC MRI without contrast acquisition is recommended. Of these options, the latter one is faster and more robust to movement. It should be noted that distortion correction with B_0_ maps or reversed phase-encode gradients (top-up correction) is problematic when subject movement occurs. This is due to both misalignment and the orientation dependence of susceptibility gradients in the main magnetic field. In these cases, the application of these methods may create additional confounding signal variations in the time series.

In addition, geometric distortions can also be exacerbated by eddy currents. While these are prominent in DWI due to the large diffusion weighting gradients, they are less relevant in DSC MRI.

In DSC MRI, blurring and point spread function (PSF) broadening are strongly influenced by the EPI echo spacing, which determines how long it takes to traverse k-space in the phase-encoding direction. Longer echo spacing increases susceptibility to field inhomogeneities and T_2_ decay *, leading to signal loss and spatial blurring that degrade image sharpness and enlarge the PSF. This impairs the ability to resolve fine anatomical or vascular structures and introduces partial volume effects, ultimately causing errors in perfusion quantification such as under- or overestimation of rCBV and rCBF due to signal mixing.

Another source of signal loss in DSC MRI arises from intravoxel dephasing caused by local magnetic field variations across tissue boundaries within a voxel [24]. This effect is amplified in sequences with large voxel sizes, where susceptibility-induced gradients lead to spins experiencing different magnetic fields and losing phase coherence over time [24]. Reducing voxel size demands higher spatial resolution, which in turn requires longer echo spacing and TE, thereby increasing T_2_* blurring and intra-voxel dephasing. As a result, the benefit of smaller voxels may be offset by increased signal degradation.

From a sequence planning perspective, shorter TE can be recommended to minimize susceptibility-induced artefacts; however, the TE is field strength dependent and should not be shorter than 30 ms for 3T MRI to obtain enough susceptibility contrast [26,27].

### 3.2. Slice Timing Misalignment

Slice timing misalignment refers to the temporal discrepancies between acquired slices. Slice timing misalignment in DSC MRI occurs because slices are acquired sequentially rather than simultaneously, typically using interleaved or sequential slice ordering. This leads to time differences between slices, meaning that contrast agent (CA) arrival is not captured at the exact same moment across all slices. This is further exacerbated when applying DSC MRI with a long repetition time (TR > 1000 ms), in which the acquired signal is confounded by physiological processes, like pulsatile flow caused by breathing and heart rate. The best way to mitigate this is to acquire multiple slices at once or to use a short TR (<500 ms), which reduces the time delay between slices [28,29].

Another option is to perform a slice timing correction to adjust the time-series data to match a reference slice. However, this is only recommended for long TRs (TR > 2000), as correction methods use interpolation, which leads to data alterations [30]. This could be either performed with the “Slice timing” tool in SPM or the “slicetimer” tool in FSL [31,32,33]. In SPM, the Slice Timing tool corrects for the time shifts using cubic spline interpolation. Required input is the slice acquisition order (e.g., interleaved or sequential) and the reference slice, to which all other slices are temporally adjusted [31]. The algorithm estimates the expected timing difference for each slice based on the time resolution and interpolates the signal to align all slices as if they were acquired simultaneously. Similarly, in FSL, the slicetimer tool performs slice timing correction using sinc interpolation to correct the time-series data for each slice, ensuring that all slices are temporally synchronized [32,33]. Required inputs are the slice acquisition order, either interleaved or sequential acquisition, and the TR value, allowing slicetimer to adjust the time-series accordingly. Both methods effectively correct slice timing offsets, reducing temporal misalignment of the contrast bolus and improving the accuracy of dynamic perfusion measurements in DSC MRI.

Single-shot EPI is the most common DSC MRI sequence being employed in clinical routine for the brain. However, multiband EPI is becoming gradually more widely used, but it has not yet reached clinical routine. Both single-shot and multiband EPI use a single TR. In order to avoid physiological artefacts, like pulsatile flow caused by breathing and heart rate, it is important to have a TR shorter than twice the resting heart rate. A normal resting heart rate can vary between 60 and 90 bpm [34,35]. Hence, a TR of <333 ms is recommended in case of single-shot or multiband EPI sequences for the brain. Multiband imaging requires enough distance between simultaneously excited slices to circumvent the signal from one slice from affecting the signal of another slice. Therefore, low multiband factors (i.e., 2 or 3) are recommended for careful separation. We must emphasize that whenever possible, TR of less than 1000 ms, optimally less than 333 ms, should be used to avoid aliasing of physiological signals, as this is the Nyquist Frequency of the average resting heart rate.

### 3.3. Image Intensity Variations (B_1_)

**Figure 3:**
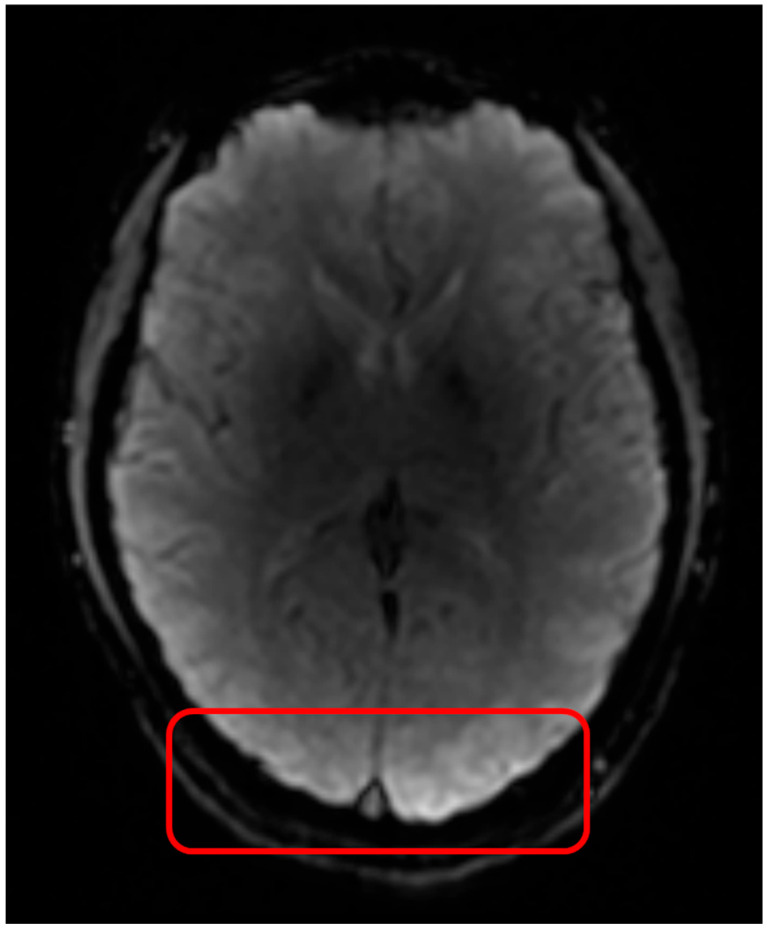
B_1_ field inhomogeneity in DSC MRI appears as spatially varying signal intensities. The red square indicates tissue hyper intensities due to B_1_ field inhomogeneities.

B_1_^+^ field inhomogeneities refer to variations or unevenness in the distribution of the transmit radiofrequency (RF) magnetic field (Figure 3), denoted as B_1_^+^, within an MRI scanner [36]. B_1_ field inhomogeneity affects all MRI acquisition types, i.e., both structural T_1_w and EPI sequences. The B_1_^+^ field is crucial because it is responsible for flipping the spins of hydrogen nuclei in the body, enabling them to be detected by the scanner to produce an image. Ideally, this field should be spatially uniform to ensure consistent flip angles and excitation of all spins across the scanned volume, but in practice, this is often not the case.

B_1_^+^ field inhomogeneities have a wide spectrum of causes. The design of the transmit RF coils and their placement relative to the subject can create areas where the B_1_^+^ field is not evenly distributed [36,37]. The presence of various tissues, organs, and other substances (like metal implants) within the human body affects the distribution of the B_1_^+^ field [38]. Factors such as the frequency of the RF pulse and the operational settings of the MRI scanner (like power and tuning) can also impact the uniformity of the B_1_^+^ field [39]. Additionally, factors such as the temperature of the scanner, the temperature of the scanner room, and subject hydration can further influence the B_1_^+^ field distribution.

One of the primary effects of B_1_^+^ inhomogeneities is the non-uniform flip angle across the MR image. To correct for B_1_^+^ inhomogeneities, the acquisition of a B_1_^+^ field map and an MP2RAGE-derived T_1_ map for correction of B_1_^+^ field inhomogeneities is currently the best option. A B_1_^+^ field map provides the actual flip angle by scaling the nominal value, while the MP2RAGE T_1_ map provides voxel-wise relaxation times [40]. Since signal intensity in GRE EPI sequences is strongly dependent on the flip angle, TR, and tissue T_1_ [22], the acquired B_1_^+^ field map and T_1_ map enable voxel-wise correction of the DSC signal. This is achieved by modeling the expected signal based on the actual flip angle using the spoiled gradient-echo signal equation [41]. The measured DSC signal is then normalized by this modeled baseline to correct for B_1_-related signal scaling. This correction ensures that observed signal changes during the passage of the contrast agent more accurately reflect true perfusion dynamics rather than variations in RF transmit efficiency.

B_1_^−^ field inhomogeneities refer to variations in the sensitivity of the RF receiver coils used to detect the MR signal. These inhomogeneities result in non-uniform signal reception across the imaging volume, often causing parts of the image to appear artificially brighter or darker, depending on the coil’s proximity and sensitivity pattern. This effect manifests as low-frequency intensity variations, which are not related to tissue properties but instead reflect differences in receiver coil sensitivity. The receive field inhomogeneity affects both structural and functional MRI and can significantly impact image quantification [42]. In DSC MRI, it may lead to spatial biases in baseline signal or perfusion estimates. To address B_1_^−^ inhomogeneities, bias field correction algorithms are commonly used. These estimate the receive sensitivity field by modeling observed intensity variations as a smooth, low-frequency multiplicative bias and correcting for it [43]. Methods such as B-spline fitting, implemented in tools like N4BiasFieldCorrection (ANTs), SPM, FSL, and others, are designed primarily to correct for the B_1_^−^ effects [31,32,43,44,45]. However, while widely validated for structural and diffusion MRI, their application in DSC MRI is still limited and requires further investigation [38]. This is because current bias field correction methods assume a static bias field across the image volume. Applying this to a time series with a contrast injection, like DSC, risks modeling and removing true signal changes as bias.

Under certain circumstances, the heating of electrical components and conductors can affect the production of B1 fields, for example, due to the transfer of heat from the gradient coil to an RF body coil during an EPI sequence. These effects are highly dependent on the scanner being used, and the potential impact on DSC-EPI should be assessed with an appropriate QA procedure.

### 3.4. Gibbs Ringing

SE DSC MRI, often used in combination with GRE in GRE-SE sequence, can suffer from Gibbs ringing artefacts. Gibbs ringing artefacts are visual distortions that appear as spurious oscillations or “rings” near the edges of sharp transitions in the image [46]. These artefacts are a common issue in MRI and originate from the mathematical process used to reconstruct images from raw data collected during the scan. More in detail, MR images are classically produced by applying an inverse Fourier transformation to data collected in the frequency domain (k-space) [46,47]. This transformation converts the frequency data into images. In practice, the MRI scanner collects a finite set of frequency data, and often the higher frequency components (which represent finer details in the image) are not fully captured. The Gibbs ringing artefact is a manifestation of the Gibbs phenomenon, which occurs in Fourier series approximations of functions with discontinuities (sharp transitions) [46,47]. When high-frequency components are missing, the sharp transitions in the image data (like the edges between different tissue types) cannot be perfectly reconstructed. This results in overshoots and undershoots at edges, appearing as rings or oscillations adjacent to these transitions. Hence, a correction for Gibbs ringing artefacts is recommended for all MRI data, including DSC MRI. If possible, different reconstruction methods could be employed that do not use the Fourier transformation, avoiding Gibbs artefacts.

The classical method for correction is the Total Variation regularization [48]. Total Variation (TV) regularization is an edge-preserving denoising technique used in image processing to reduce artefacts while maintaining sharp edges. TV regularization is particularly effective against Gibbs ringing by applying piecewise smoothness in the image while avoiding excessive smoothing of edges. MRtrix has the “mrdegibbs” tool to correct for Gibbs ringing; however, this has only been validated for anatomical and diffusion MRI [49,50]. With respect to DSC MRI, the application of Gibbs ringing corrections remains poorly investigated. This is mainly because DSC MRI captures dynamic signal fluctuations caused by the passage of a contrast agent, leading to rapid T_2_w* changes over time. Since TV regularization assumes a piecewise-smooth signal model, applying it to DSC MRI could unintentionally smooth out important contrast-induced variations, distorting perfusion quantification. Additionally, Gibbs artefacts in DSC MRI may exhibit dynamic variations, rather than remaining spatially stable as in static imaging, potentially reducing the effectiveness of TV-based corrections or introducing unwanted temporal inconsistencies and a significant ‘whitening’ of the noise spectrum (see Section 3.6). Hence, future research may need to explore adaptive or hybrid approaches that incorporate both spatial and temporal constraints to preserve the integrity of perfusion dynamics while effectively mitigating Gibbs artefacts. Other possibilities to reduce Gibbs artefacts are related to scan parameter optimization, like increasing the matrix size, reducing the FoV, or reducing the bandwidth [51,52].

### 3.5. Subject Motion

**Figure 4:**
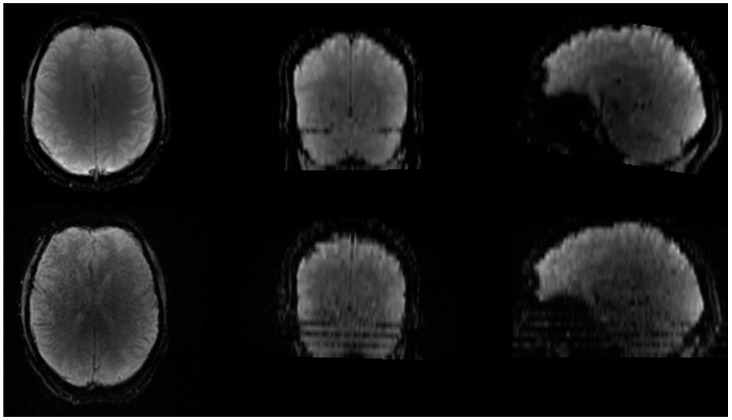
Subject motion causes image blurring and may lead to stripes in the phase encoding direction. Top panel is DSC acquisition without motion, bottom panel is DSC acquisition with motion.

**Figure 5:**
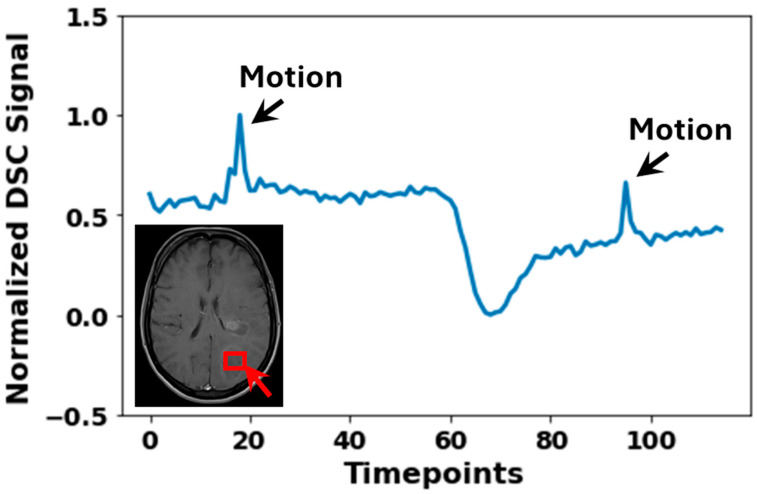
Normalized DSC MRI signal of a 3 × 3 × 3 mm^3^ cube region (indicated by the red box), demonstrating the presence of motion artefacts. Distinct peaks labeled “Motion” indicate signal disruptions caused by patient movement. Normalization was the division of observed signal intensity by max observed signal intensity.

Subject motion is a common issue in MRI scans, particularly in modalities that require dynamic image acquisitions, such as fMRI and DSC MRI. While motion can introduce artefacts (Figure 4) and affect the accuracy of perfusion measurements, the rapid EPI acquisitions allow for some correction possibilities through rigid body alignment [29,53,54]. The motion correction step ensures that any artefacts due to head movement are minimized, improving the consistency and reliability of the measurements across different scans. Prior to the application of motion correction, the first determination of motion within the data should be made. This motion determination is performed using visual inspection of both the DSC images themselves (i.e., does the brain location change during acquisition) and by plotting the time–signal–intensity curve (Figure 5). Within the time–signal–intensity curves, motion is often visualized as a sharp peak. When assessing the motion between the images, the first acquired image should be used as a reference to which the other images are compared for motion determination. If the observed motion is larger than the voxel size of the images, motion should be corrected for. If motion is smaller than the voxel size of the images, motion should not be corrected for. This is because the application of motion correction uses interpolation to reach an agreement (i.e., similar voxels in the surrounding regions). Such interpolation induces noise in the images, which may affect the parameter extraction. However, when the motion is larger than the voxel size, the motion itself introduces more noise than the interpolation, and hence, motion correction should be applied. Aside from translation, motion rotation is also important to consider. If rotational motion is smaller than the in-plane angular resolution implied by the voxel dimensions and brain size, motion correction is generally not necessary. This is because the minor angular displacement typically results in sub-voxel misalignments that do not substantially alter the spatial correspondence across time points. Correcting for such small rotations requires interpolation, which can introduce spatial smoothing and temporal noise, potentially degrading the accuracy of perfusion metrics. However, when rotational motion exceeds this threshold, causing misalignment of structures beyond the voxel size, uncorrected motion becomes a greater source of error than interpolation. In these cases, motion correction should be applied to preserve the consistency of anatomical locations over time and to ensure accurate extraction of perfusion parameters. Measurement of the motion can be performed on a very broad scale of image viewing software, like DICOM viewers or Vinci.

In the case of motion correction in DSC MRI, the images prior to bolus injection are commonly used as a reference. This, however, can be problematic due to vastly differing image contrasts between pre and post bolus, and lead to the introduction of artificial motion artefacts due to misalignment. For DSC MRI, methods that are optimized for aligning images with varying intensities and noise structures, like normalized mutual information, might be a better approach than methods that perform best when image contrast remains stable (i.e., within modality motion correction), like normalized cross correlation [31]. Nevertheless, this can still lead to misalignments during the contrast bolus passage if not meticulously monitored using visual inspection. Currently, no comparison between the motion correction methods for DSC MRI has been performed, and therefore remains debatable which method might be preferred.

### 3.6. Noise

**Figure 6:**
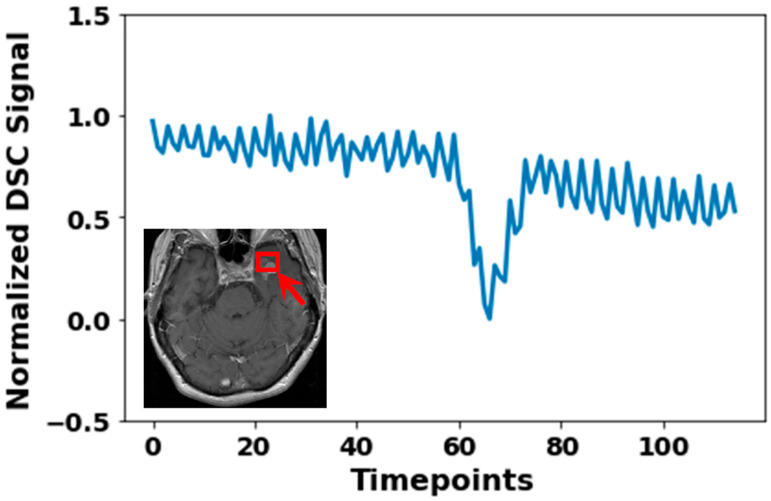
Normalized DSC MRI signal of a 3 × 3 × 3 mm^3^ cube region (indicated by the red box), illustrating substantial noise artefacts. The fluctuating pattern indicates physiological noise. Normalization was the division of observed signal intensity by max observed signal intensity.

DSC MRI suffers not only from thermal and electronic noise, but also physiological noise and noise originating in the reconstruction process. Thermal and electronic noise are caused by random electron motion in MRI hardware (i.e., RF coils, resistors, and amplifiers) and have a Gaussian distribution [55,56]. Physiological noise is due to breathing, heartbeat, and pulsatile blood flow (Figure 6) [29,57]. This could be circumvented by either measuring physiological data (i.e., heart rate and respiration) to correct for the effects of physiological data on the DSC signal or by using a TR smaller than twice the resting heart rate (i.e., 333 ms at a resting heart rate of 90 bpm). When physiological data is measured, the physiological noise can be corrected for with a wide range of tools, including the PhysIO toolbox for SPM [58], the 3dretroicor toolbox for AFNI [59], and the physiological noise modeling toolbox for FSL [60]. Per sequence, a phase and magnitude image are generated. Phase images contain information about magnetic field variations, motion, and flow effects. Magnitude images represent the signal intensity based on tissue properties. DSC MRI relies predominantly on magnitude images due to their direct correlation with susceptibility-induced signal changes. Magnitude images suffer from Rician noise, which is due to the mathematical procedures during the conversion of the MRI signal into magnitude images [61,62]. At low signal-to-noise ratio (SNR) (SNR < 2), the Rician noise approaches a Rayleigh distribution, whereas at SNR > 2, Rician noise approximates a Gaussian distribution [61]. Hence, low SNR regions like CSF or close to susceptibility artefacts may be dominated by Rician noise, significantly complicating analysis. The noise structure in phase images always remains Gaussian. Currently, there are no validated methods to correct for the Rician noise.

Within DSC MRI, throughout the entire signal intensity time curve, noise is present. Upon arrival of the contrast agent bolus, the signal intensity decreases, but the noise remains constant [63]. Hence, SNR decreases after bolus arrival. Upon parameter estimation, the signal intensity time curves are often converted to contrast concentration–time curves. While the noise in the signal intensity time curves is independent of signal amplitude, noise in contrast concentration time curves is amplitude-dependent [63]. Because signal intensity and contrast concentrations are non-linearly (i.e., log-transform) related, the transformation alters the noise characteristics, making the noise variance dependent on the signal amplitude [63,64]. Specifically, lower signal intensities (which correspond to higher contrast agent concentrations) result in higher noise variance after transformation. This amplitude-dependent noise can lead to increased uncertainty in the estimation of perfusion parameters derived from concentration time curves.

Enhancing parameter estimation robustness is an ongoing topic of investigation [64,65,66,67,68,69,70]. However, these studies normally do not aim to reduce the noise, but rather aim to develop parameter estimation methods that are robust to noise.

Denoising is recommended to be performed early in the pre-processing chain to prevent whitening of the noise spectrum, which means that the different noises can no longer be distinguished from each other. However, the application of denoising algorithms to DSC MRI data remains poorly investigated and thus still requires further investigation.

### 3.7. Data Quality Assurance

The first step of quality assurance is that a medical physicist regularly makes phantom scans in order to ensure the consistency, accuracy, and reliability of imaging measurements. The NVKF (Nederlandse Vereniging voor Klinische Fysica) quality control guidelines for radiological equipment recommend performing phantom scans once every two weeks following the installation of an MRI scanner. This frequency is advised to establish a baseline and monitor potential early performance deviations. Additionally, after periodic maintenance, phantom scanning is crucial, as hardware adjustments or replacements (e.g., gradient coils, RF coils, or software updates) may alter imaging parameters, affecting image quality and quantitative reproducibility. Once baseline performance has been established, the frequency for phantom scans can be reduced to approximately once per month, balancing practical feasibility with rigorous quality assurance.

The phantom scans can be performed with, for instance, the American College of Radiology (ACR) MRI phantom [71]. This cylindrical phantom contains structures designed to evaluate several key imaging quality parameters, although not all, as it is a fixed phantom and does not replicate dynamic physiological conditions of the body. Using the ACR phantom, geometric distortions (B_0_ field inhomogeneities), signal losses related to spatial resolution, image intensity nonuniformity (B_1_ field inhomogeneities), and Gibbs ringing artefacts can be effectively assessed. However, dynamic artefacts such as slice timing misalignments, motion-related distortions, and physiological noise (e.g., fluctuations due to heartbeat and respiration) cannot be evaluated with this phantom. Nevertheless, regular use of the ACR MRI phantom is highly valuable, as it ensures consistent baseline image quality critical for reliable perfusion measurements.

By performing periodic quality control (QC) scans, a trendline of various image quality parameters is established, allowing for the early detection of gradual or sudden hardware degradation. This approach ensures that potential issues affecting signal uniformity, spatial accuracy, B_0_ and B_1_ field homogeneity, or gradient performance are identified before they compromise clinical imaging. If quality control scans indicate deviations from expected performance, the vendor (i.e., Siemens, Philips, GE) is often consulted to evaluate the system’s functionality through diagnostic tests to verify whether the MRI scanner operates within specifications. If a hardware component is found to be faulty, the respective component is either repaired or replaced to restore scanner performance. Additionally, if a scanner is found to be temporarily unsuitable for certain applications due to performance issues, the medical physics team assesses the scanner’s reliability for other specific imaging tasks.

With phantom scans ensuring a baseline for the technical state of the system that translates into a consistency of image quality parameters, the next step is to ensure the proper scanning protocols are in place. Optimizing scan parameters can reduce the degree to which artefacts manifest in the final image. In addition, MRI technicians should receive proper training to deliver constant quality and assess if the image is of sufficient quality.

Next, after each pre-processing step, the images should be carefully visually inspected to determine whether the images have been properly pre-processed. This can be performed by loading the DSC images and scrolling through the images to determine image abnormalities. Abnormalities due to image processing are generally easy to determine as they result in distortions that alter the spatial integrity and anatomical representation of the image. Therefore, the unprocessed images and the images of the previous pre-processing step should be loaded as a frame of reference. If the distortions are not present on both the unprocessed and during the previous pre-processing step, then this means that it has been introduced during the execution of the current pre-processing step. To mitigate the distortions, different settings of the corresponding pre-processing step could be applied.

## 4. Discussion

DSC MRI is used in clinical routine and is considered a quantitative MRI method. However, there is a lack of standardization of the pre-processing of DSC MRI. The absence of standardized pre-processing in DSC MRI affects diagnostic and research reliability. In the worst-case scenario, sub-optimal pre-processing might lead to both erroneous diagnostics and scientific findings. With respect to DSC MRI artefacts, we identified six main categories: geometric distortions, slice-timing misalignment, image intensity variations, Gibbs ringing, motion, and noise.

Noteworthy is that three types of artefacts can only be circumvented by acquiring more data or by improving the DSC MRI acquisition: geometric distortions related to the B_0_ field, geometric distortions due to partial volume effects, and physiological noise. Geometric distortions related to the B_0_ field can only be compensated for when either a B_0_ field map or a reversed phase-encoding acquisition is acquired [19,20,24,25]. High-temporal resolution in EPI has led to poor spatial resolution, often resulting in significant partial volume effects and suboptimal reslicing outcomes [24,72,73]. Modern acceleration techniques (i.e., parallel imaging, compressed sensing, simultaneous multi-slice) can facilitate the acquisition of isotropic voxels, which help reduce partial volume effects. Among these, SMS is particularly effective in increasing the number of slices acquired per unit time and thus has the greatest impact on achieving isotropic resolution within a feasible TR. While parallel imaging and compressed sensing can reduce EPI readout duration and support higher in-plane resolution, their impact on overall TR is more modest, as the total number of slice excitations remains unchanged. Moreover, implementing compressed sensing in 2D EPI is technically challenging due to the nonlinear phase evolution it can introduce in off-resonance conditions. Furthermore, for physiological artefacts, which are artefacts introduced due to natural biological processes (i.e., breathing, heart rate), the TR of the image volume also plays a crucial role [29,57]. If the TR is slower than twice the heart rate (>1000 ms), aliasing errors that cannot be corrected might occur. A TR of less than 333 ms or recording physiological data is advised to mitigate this risk [29,57]. The use of a short TR can also obviate the need for a slice timing misalignment correction, as the physiological processes are significantly slower than the sampling rate.

One of the major problems related to DSC MRI pre-processing is that the effectiveness of these methods also varies depending on the MRI scanner field strength and acquisition parameters, limiting the universal applicability [74,75]. Higher magnetic field strengths (≥3T) introduce greater susceptibility effects, leading to more pronounced geometric distortions in DSC MRI due to increased B_0_ inhomogeneity [76]. This makes field map-based distortion correction more critical at high fields. Additionally, higher field strengths exacerbate B_1_ inhomogeneity, necessitating bias field correction [77]. However, higher fields also provide higher SNR, which may reduce the need for aggressive denoising algorithms. Conversely, at lower field strengths (1.5T or below), thermal noise becomes more dominant [78]. Acquisition settings, including TR, TE, voxel size, EPI readout duration, and slice acquisition order, significantly affect the applicability of correction methods. Longer TE values exacerbate susceptibility distortions, increasing the need for geometric correction methods. Longer TR values introduce greater slice timing misalignment, making slice-timing correction essential [30]. The quality of the raw data also heavily influences the success of pre-processing, with suboptimal acquisition settings (e.g., poor resolution or long TR) leading to artefacts that are challenging to correct.

In DSC MRI processing, the order of corrections is critical to preserve the integrity of the time signal intensity curves used for perfusion quantification. First, motion correction should be applied to align volumes across time, ensuring that the same anatomical location is sampled consistently throughout the dynamic series. This alignment is essential before applying any spatial corrections, as uncorrected motion can distort the mapping of distortion fields or physiological noise models. Second, correction for geometric distortions due to B_0_ field inhomogeneities (e.g., using field maps or topup techniques) should be performed on the motion-corrected data to accurately unwarp spatial deformations, particularly in EPI-based acquisitions where susceptibility artefacts are pronounced. Lastly, physiological noise correction, such as modeling or filtering out pulsatile flow, respiratory fluctuations, or global signal drifts, should be applied, as these typically rely on stable spatial and temporal alignment to effectively isolate non-neuronal signal fluctuations. This sequence, motion, then distortion, then physiological correction, minimizes error propagation in the final perfusion maps.

For DWI pre-processing, denoising, corrections for Gibbs ringing, geometric distortions, Eddy currents, motion, and B1 bias field are well validated and have become standard practice. DSC MRI pre-processing is inherently more complex due to its temporal component, which makes many common spatial processing steps less straightforward and more prone to introducing artifacts that bias quantitative perfusion measurements.

Currently, pre-processing methods of DSC MRI are rarely available on the scanner, requiring offline processing. This significantly delays the availability of processed images to the radiologist and requires in-house specialists in image processing. Hence, pre-processing of DSC MRI would find poor translation into routine clinical settings. This is comparable to DWI, for which proper pre-processing is performed offline and is therefore not used in routine clinical care. The developments of installing software containers on the scanners enable direct on-scanner image pre-processing. However, this development is only for state-of-the-art MRI scanners and hence not widely accessible. Only when studies show the impact of proper pre-processing on diagnostic accuracy, DSC pre-processing might reach routine clinical applications.

The limitations of this article are that emerging techniques for pre-processing have not been considered, as the primary focus was on currently established pre-processing steps, potentially overlooking innovative solutions. Furthermore, the study focuses on specific artefacts and does not address broader issues, such as variability in contrast agent delivery or patient physiological differences, highlighting the need for further research and validation. Important aspects to consider related to the bolus injection are the bolus concentration, speed of injection, needle width, tube width, venous access (avoiding clamping of intravenous line), saline flushing directly post-bolus injection, and the power of the injector. These aspects are thoroughly assessed and described previously [75]. Another source of bias on DSC-derived perfusion parameters is contrast agent leakage [12,13,75,79]. This can create major artefacts and biases in DSC MRI. However, as this is a biological phenomenon unrelated to image quality, we did not address this. Nonetheless, leakage has been thoroughly addressed previously [12,13,75,79].

Standardizing pre-processing steps such as correcting geometric EPI distortions, B_1_ field inhomogeneities, Gibbs ringing artefacts, slice timing, subject motion, physiological artefacts, and noise might greatly improve the accuracy and consistency of DSC MRI. This benefits both clinical and research applications. However, it is crucial to assess the efficacy of these steps, as improper processing can introduce new artefacts or distort data, necessitating visual inspections against original data to ensure artefact sources are correctly identified. Further validation is needed for many of these pre-processing steps to ensure their effectiveness in DSC MRI analysis. Yet there is a general consensus regarding the application of corrections for B_0_ field inhomogeneities, slice-timing misalignments if TR > 2000 ms, motion, and physiological noise, including pulsatile flow. Due to the lack of validated DSC pre-processing techniques, it would be highly beneficial to the community to establish collaboration between institutions or a working group to standardize and validate DSC pre-processing protocols.

## Figures and Tables

**Table 1:** Overview of DSC MRI artefacts and validation status for corresponding pre-processing methods. Excellent means that literature has shown that the method works well; poor means that this has scarcely been investigated for DSC MRI.

Artefact	Pre-Processing Methods/Tools	Practical Applicability Based on Expert Opinion	Validation Status
Geometric distortions (B_0_)	Non-rigid spatial transformations based on scan with opposite phase encoding (FSL Topup) or using a B_0_ fieldmap (SPM Field map Toolbox)	Useful	Extensive (fMRI literature)
Image intensity variations (B_1_)	Modeling the low-frequency bias in order to correct for it	Potentially useful	Poor
Gibbs ringing artefacts	Subvoxel shifts/TV-regularization in MRtrix	Potentially useful with high-resolution data	Poor
Slice timing misalignment	Several tools and scripts, i.e., SPM Slice timing tool, FSL slicetimer	Useful if high TR	Good for high TR
Physiological artefacts	SPM PhysIO Toolbox, AFNI 3dretroicor, or FSL physiological noise modeling toolbox	Necessary if high TR	Good
Subject motion	Rigid body registration incl. mutual information, many tools, including SPM and FSL	Necessary if motion is present	Extensive
Noise	Several	Questionable	Extensive, but mixed results.

TV-regularization = Total Variation regularization, EPI = echo planar imaging, TR = relaxation time, FSL = FMRIB software library, SPM = statistical parametric mapping, AFNI = analysis of functional neuroimages.
